# Cardiac Catheterizations in Patients With Prior Coronary Bypass Surgery: Impact of Access Strategy on Short-Term Safety and Long-Term Efficacy Outcomes

**DOI:** 10.1177/0003319720987351

**Published:** 2021-01-19

**Authors:** Frederik T. Groenland, Jeroen M. Wilschut, Stijn C. van den Oord, Isabella Kardys, Roberto Diletti, Peter P. de Jaegere, Felix Zijlstra, Joost Daemen, Nicolas M. Van Mieghem, Wijnand K. den Dekker

**Affiliations:** 1 Department of Cardiology, Thoraxcenter, 6993 Erasmus University Medical Center, Rotterdam, the Netherlands; 2 Department of Cardiology, Radboud University Medical Center, Nijmegen, the Netherlands

**Keywords:** coronary angiography, coronary artery bypass graft surgery, percutaneous coronary intervention, transfemoral access, transradial access, vascular complications

## Abstract

Little data are available on access strategy outcomes for cardiac catheterizations in patients with prior coronary artery bypass graft surgery (CABG). We investigated the effect of transradial access (TRA) and transfemoral access (TFA) on short-term major vascular complications (MVC) and long-term major adverse cardiovascular events (MACE). In this single-center, retrospective cohort study, 1084 patients met our inclusion criteria (TRA = 469; TFA = 615). The cumulative incidence for the primary safety endpoint MVC at 30 days (a composite of major bleeding, retroperitoneal hematoma, dissection, pseudoaneurysm, and arteriovenous fistula) was lower with TRA (0.7% vs 3.0%, *P* < .01) and this difference remained significant after propensity score adjustment (odds ratio: 0.24; 95% CI, 0.07-0.83; *P* = .024). The cumulative incidence for the primary efficacy endpoint MACE at 36 months (a composite of all-cause mortality, myocardial infarction, stroke, and urgent target vessel revascularization) was 28.6% with TRA and 27.6% with TFA, respectively. Kaplan-Meier curves showed no difference for the primary efficacy endpoint (*P* = .65). Contrast use (mL) was significantly lower with TRA (130 [100-180] vs 150 [100-213], *P* < .01). In conclusion, in patients with prior CABG, TRA was associated with significantly fewer short-term MVC and contrast use, but not with a difference in long-term MACE, compared with TFA.

## Introduction

Transradial access (TRA) is the first choice for coronary angiography (CA) and percutaneous coronary intervention (PCI) in current clinical practice.^1–3^ Several randomized controlled trials (RCTs) and meta-analyses have shown that TRA is superior to transfemoral access (TFA) in terms of vascular complications and major bleeding.^4–7^ Furthermore, most of these studies did not observe differences in short-term and 1-year major adverse cardiovascular events (MACE).^4–6,8^

Whether this also holds true for patients with prior coronary artery bypass graft surgery (CABG) is unclear. Studies comparing access site strategy in these patients are limited, despite the fact that CA and PCI are regularly performed in this population. Consequently, patients with prior CABG still have a high likelihood of TFA over TRA.^9–11^ In the RIVAL (radial versus femoral access for CA and intervention in patients with acute coronary syndromes) and MATRIX (radial versus femoral access and bivalirudin versus unfractionated heparin in invasively managed patients with acute coronary syndrome) study, prior CABG was not an exclusion criterion.^4,5^ Nevertheless, few patients with a history of CABG were included and data were not analyzed separately. Some observational studies did compare TRA with TFA in these patients. They showed lower rates of vascular and bleeding complications in the TRA group, but no differences in MACE rate up to 1-year.^10–14^ The only RCT so far did not find differences in MACE or vascular complications.^15^

To our knowledge, studies comparing the impact of access strategy on long-term clinical outcomes are not available for patients with prior CABG. Furthermore, data on vascular complications for this subset of patients undergoing CA and PCI are still limited.

The aim of this study was to investigate the effect of TRA and TFA on short-term major vascular complications (MVC) and long-term MACE.

## Materials and Methods

### Study Design and Patient Population

We conducted a single-center, retrospective cohort study to compare TRA and TFA for cardiac catheterization in CABG patients. From July 2010 to December 2017, 1106 consecutive patients with a history of CABG underwent CA or PCI in our tertiary care institution. Patients with a brachial or ulnar arterial access, a radial artery used as bypass graft and a heart transplant after CABG were excluded. If multiple procedures were performed during the study period, patients were included at their first CA or PCI. The Ethics Committee of the Erasmus Medical Center approved the research proposal and concluded that the study was not subject to the Dutch Research on Humans Subjects Act, renouncing the need for patient informed consent.

### Procedure Description

Multiple interventional cardiologists performed the CA and PCI procedures during the study period. The selection for TRA or TFA was made at the operator’s discretion. Arterial access was obtained via the right or left radial artery in the TRA group and via the right or left common femoral artery in the TFA group. If arterial crossover was performed, the initial access site was decisive for placement in the TRA or TFA group. Procedural success for PCI was defined as successful stent placement, unless it was stated that the lesion was only treated with balloon inflation. Hemostasis was acquired by a transradial compression device in the TRA group and by an arterial closure device or by manual compression in the TFA group. All procedures were performed according to the standard of practice at that time and periprocedural medication was given in line with treatment protocols, including heparin and in case of TRA a cocktail of verapamil and nitroglycerin. Patients undergoing PCI were loaded with aspirin and a P2Y12 inhibitor (clopidogrel, prasugrel, or ticagrelor).

### Study Endpoints

To assess for outcome differences between TRA and TFA, we determined a primary safety endpoint and a primary efficacy endpoint. The primary safety endpoint was defined as MVC, a composite of non-CABG-related major bleeding, retroperitoneal hematoma, dissection, pseudoaneurysm, and arteriovenous fistula at 30 days after the procedure. A non-CABG-related major bleeding was specified as a “Bleeding Academic Research Consortium” (BARC) type 3 or 5 bleeding.^16^ The primary efficacy endpoint was defined as long-term MACE, a composite of all-cause mortality, myocardial infarction (MI), stroke, and urgent target vessel revascularization (UTVR). We performed censoring at 36 months, as at least 50% of follow-up was available for the total study population. Stroke and MI were diagnosed by the treating physician according to standard guidelines at the time of event, and UTVR was described as a repeat PCI of the same coronary artery in patients with unstable angina, non-ST-elevation MI, and ST-elevation MI. The individual components of the primary safety and efficacy endpoint were defined as secondary safety and efficacy endpoints. Furthermore, major bleeding subtypes (BARC type 3 A/B/C, transfusion, type 5 A/B), minor and overall bleeding at 30 days were examined. Minor bleeding was defined as a BARC type 1 or 2 bleeding (16). Overall bleeding included both non-CABG-related major bleeding and minor bleeding.

### Data Collection

We extracted baseline and procedural data from the hospital’s electronic medical record system, as well as data on MVC and bleeding. The occurrence of MACE since the procedure was assessed for each patient individually between May and July 2019. Data on mortality were obtained from the municipal civil registry. If follow-up data were not available in the medical records, we collected information on patient follow-up using a written questionnaire or telephone survey. Only if necessary and after patient’s permission, the treating physician was contacted to complete data collection.

### Statistical Analysis

Continuous variables were presented as medians (25th-75th percentiles) and compared using the Mann-Whitney *U* test. Categorical variables were expressed as percentages (%) and compared with Pearson χ^2^ test or Fisher exact test, as appropriate. To investigate differences in efficacy endpoints between TRA and TFA, Kaplan-Meier curves were drawn and log-rank tests were performed. Patients were followed-up until the time of the event or until the date of last contact, at which time point they were censored. To investigate differences in all safety outcomes and bleeding between both access sites, Pearson χ^2^ test or Fisher exact test was used. Subsequently, logistic regression models with complete case analysis (n = 1043) were used to determine whether TRA was associated with MVC and bleeding. First, we performed univariable analysis. Results were expressed as odds ratios (ORs) with 95% CIs. To perform multivariable analysis, we used propensity score (PS) adjustment because of the limited number of available events per variable. The PS was calculated for each patient using logistic regression with access site (TRA or TFA) as the dependent variable. We selected the following clinically relevant independent variables in the PS model: age, sex, hypertension, hypercholesterolemia, diabetes, family history, current smoking, prior stroke, prior MI, prior PCI, peripheral artery disease, chronic kidney disease, CABG anatomy known, CA or PCI within 48 hours post-CABG, type of graft(s) used, ST-elevation MI, PCI performed, sheath size (6F), type of vessel treated, lesion class B2 or C, implanted stents, total stent length, procedure time, fluoroscopy time, radiation exposure (dose area product), use of glycoprotein inhibitor, and use of thienopyridine derivate. We then performed multivariable logistic regression with the calculated PS and TRA as independent variables. Adjusted ORs with 95% CIs were thus obtained. SPSS version 25.0 was used for statistical analyses. A 2-sided *P* < .05 was considered significant.

## Results

### Baseline Characteristics

A total of 1084 patients with a history of CABG who underwent CA or PCI were included in the study. Of the 1106 consecutive patients who were evaluated, 22 were excluded due to brachial arterial access (n = 4), ulnar arterial access (n = 4), heart transplant after CABG (n = 8), and a radial artery used as a bypass graft (n = 6). The baseline characteristics are shown in Table 1. Arterial access was obtained through TRA in 469 patients and through TFA in 615 patients. Patients in the TRA group were significantly older than those in the TFA group. Prevalence of cardiovascular risk factors was high in both groups, but did not differ significantly, except for a family history of cardiovascular disease. Patients in the TFA group had more often ST-elevation MI at clinical presentation or CABG in the previous 48 hours. Furthermore, graft anatomy was known before cardiac catheterization in the great majority of patients and did not differ significantly between TRA and TFA.

**Table 1.** Baseline Characteristics.^a^

Variable	Total (n = 1084)	TRA (n = 469)	TFA (n = 615)	*P*
Age (years)	72 [65-77]	72 [66-78]	71 [65-77]	**.018**
Male	853/1084 (78.7)	370/469 (78.9)	483/615 (78.5)	.89
Hypertension	876/1082 (81.0)	388/468 (82.9)	488/614 (79.5)	.16
Hypercholesterolemia	868/1082 (80.2)	383/468 (81.8)	485/614 (79.0)	.24
Diabetes	388/1082 (35.9)	163/468 (34.8)	225/614 (36.6)	.54
Family History	349/1082 (32.3)	135/468 (28.8)	214/614 (34.9)	**.036**
Current Smoker	104/1082 (9.6)	39/468 (8.3)	65/614 (10.6)	.21
Prior Stroke	172/1082 (15.9)	75/468 (16.0)	97/614 (15.8)	.92
Prior MI	516/1082 (47.7)	211/468 (45.1)	305/614 (49.7)	.13
Prior PCI	440/1082 (40.7)	183/468 (39.1)	257/614 (41.9)	.36
Peripheral artery disease	267/1082 (24.7)	110/468 (23.5)	157/614 (25.6)	.44
Chronic kidney disease	283/1083 (26.1)	120/469 (25.6)	163/614 (26.5)	.72
Clinical presentation				
Stable angina	405/1084 (37.4)	179/469 (38.2)	226/615 (36.7)	.63
Unstable angina/NSTEMI	392/1084 (36.2)	178/469 (38.0)	214/615 (34.8)	.28
STEMI	130/1084 (12.0)	34/469 (7.2)	96/615 (15.6)	**<.01**
Other	157/1084 (14.5)	78/469 (16.6)	79/615 (12.8)	.08
CABG				
Time since (years)	12 [4-18]	12 [5-19]	12 [4-18]	.16
Graft anatomy known	1051/1084 (97.0)	457/469 (97.4)	594/615 (96.6)	.42
In patients with STEMI	118/130 (90.8)	31/34 (91.2)	87/96 (90.6)	.92
CA/PCI < 48 hours	73/1084 (6.7)	13/469 (2.8)	60/615 (9.8)	**<.01**
LIMA used	920/1084 (84.9)	399/469 (85.1)	521/615 (84.7)	.87
RIMA used	116/1084 (10.7)	61/469 (13.0)	55/615 (8.9)	**.032**
SVG used	974/1084 (89.9)	417/469 (88.9)	557/615 (90.6)	.37

Abbreviations: CA, coronary angiography; CABG, coronary artery bypass graft; LIMA, left internal mammary artery; MI, myocardial infarction; NSTEMI, non-ST-elevation myocardial infarction; PCI, percutaneous coronary intervention; RIMA, right internal mammary artery; STEMI, ST-elevation myocardial infarction; SVG, saphenous vein graft; TFA, transfemoral access; TRA, transradial access. Bold values highlight the statistical significance. ^a^ Values are median (interquartile range) or n (%).

### Procedural Characteristics

The procedural characteristics are shown in Table 2. Percutaneous coronary intervention was performed in 699 (64.5%) patients and CA was performed in the remaining 385 (35.5%) cases. Percentage of PCI and success rates for PCI were comparable for both the TRA and TFA group. Native coronary arteries were more often treated through TRA, while grafts were more often treated through TFA. Access site crossover occurred significantly more often from TRA to TFA, than vice versa. Use of a 5F sheath was more common with TRA, while use of a 6F sheath was more common with TFA. Use of a 7F sheath did not differ significantly between both access sites. The use of only aspirin after the procedure was less in the TRA group, while use of triple anticoagulation therapy was more in the TRA group. Procedure and fluoroscopy time (minutes) were comparable for both groups, but radiation exposure (cGycm^2^) and contrast use (mL) were significantly lower in the TRA group.

**Table 2.** Procedural Characteristics.^a^

Variable	Total (n = 1084)	TRA (n = 469)	TFA (n = 615)	*P*
Access site crossover	35 (3.2)	31 (6.6)	4 (0.7)	**<.01**
Sheath size				
5F	20 (1.8)	20 (4.3)	0 (0.0)	**<.01**
6F	1046 (96.5)	445 (94.9)	601 (97.7)	**.018**
7F	18 (1.7)	4 (0.8)	14 (2.3)	.07
PCI performed	699 (64.5)	309 (65.9)	390 (63.4)	.40
Procedural success	665 (95.1)	297 (96.1)	368 (94.4)	.28
Vessel treated				
Native	361 (51.6)	178 (57.6)	183 (46.9)	**<.01**
Graft	202 (28.9)	76 (24.6)	126 (32.3)	**.025**
Multiple	136 (19.5)	55 (17.8)	81 (20.8)	.33
Lesion class^b^				
Class A	9 (1.3)	6 (1.9)	3 (0.8)	.19
Class B1	63 (9.0)	32 (10.4)	31 (7.9)	.27
Class B2	145 (20.7)	67 (21.7)	78 (20.0)	.59
Class C	482 (69.0)	204 (66.0)	278 (71.3)	.14
Implanted Stents	1 [1-2]	1 [1-2]	1 [1-2]	.20
Stent length (mm)	27 [16-46]	27 [16-45]	27 [16-48]	.61
Stent used	651 (93.1)	290 (93.9)	361 (92.6)	.50
Drug-eluting stent	638 (98.0)	285 (98.3)	353 (97.8)	.42
Bare Metal Stent	7 (1.1)	3 (1.0)	4 (1.1)	1.00
Other	6 (0.9)	2 (0.7)	4 (1.1)	.70
Embolic protection device	49 (4.5)	21 (4.5)	28 (4.6)	.95
Procedure time (minutes)	61 [43-83]	61 [45-83]	61 [42-83]	.67
Fluoroscopy time (minutes)	15 [9-24]	15 [10-23]	15 [9-24]	.51
Radiation exposure (cGycm^2^)	5186 [3168-8805]	4608 [2974-7274]	5828 [3473-9711]	**<.01**
Contrast (mL)	150 [100-200]	130 [100-180]	150 [100-213]	**<.01**
Glycoprotein inhibitor	55 (5.1)	17 (3.6)	38 (6.2)	.60
Medication at discharge				
Aspirin only	190 (17.5)	68 (14.5)	122 (19.8)	**.022**
Dual antiplatelet therapy	759 (70.0)	332 (70.8)	427 (69.4)	.63
Triple anticoagulation therapy	122 (11.3)	67 (14.3)	55 (8.9)	**<.01**

Abbreviations: PCI, percutaneous coronary intervention; TFA, transfemoral access; TRA, transradial access.

Bold values highlight the statistical significance. ^a^ Values are median (interquartile range) or n (%).

^b^ Lesion class of the most severe lesion, if multiple were treated.

### Trends in TRA Use and Data Collection

**Figure 1.**
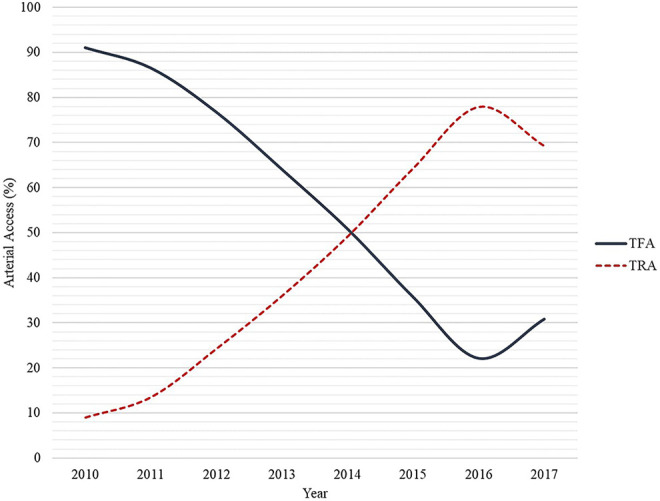
Access site for coronary angiography and percutaneous coronary intervention in patients with prior coronary artery bypass graft surgery; use of TFA and TRA over time (2010-2017). TFA indicates transfemoral access; TRA, transradial access.

In 2015, TRA became the preferred choice for CA and PCI in patients with a history of CABG at our center (Figure 1). During the study period, proportion TRA increased significantly over the years compared with TFA (*P* for trend <.01). TRA expanded from 9% in 2010 to 69.2% in 2017. In 2016, 77.9% of the procedures were performed through TRA. As shown in Tables 1 to 3, baseline and procedural characteristics were largely complete (>95%) and loss to follow-up percentages were low at 30 days (<3%).

### Safety Endpoints and Bleeding

As shown in Table 3, MVC occurred significantly less often in the TRA group, as did major bleeding. Aneurysm did not differ significantly between the two groups. For the other secondary safety outcomes, no statistical tests were performed because of the low numbers of events. Occurrence of minor and overall bleeding was significantly lower in the TRA group.

**Table 3.** Major Vascular Complications and Bleeding at 30 Days.^a^

Outcome	Total (n = 1084)	TRA (n = 469)	TFA (n = 615)	*P*
Major vascular complications	21/1061 (2.0)	3/458 (0.7)	18/603 (3.0)	**<.01**
Major bleeding^b^	18/1061 (1.7)	2/458 (0.4)	16/603 (2.7)	**<.01**
Type 3a	10/1061 (0.9)	2/458 (0.4)	8/603 (1.3)	.14
Transfusion	4/1061 (0.4)	1/458 (0.2)	3/603 (0.5)	.64
Type 3b	5/1061 (0.5	0/458 (0.0)	5/603 (0.8)	.07
Type 3c	1/1061 (0.1)	0/458 (0.0)	1/603 (0.2)	^c^
Type 5a	1/1061 (0.1)	0/458 (0.0)	1/603 (0.2)	^c^
Type 5b	1/1061 (0.1)	0/458 (0.0)	1/603 (0.2)	^c^
Aneurysm	3/1061 (0.3)	0/458 (0.0)	3/603 (0.5)	.26
Dissection	2/1061 (0.2)	1/458 (0.2)	1/603 (0.2)	^c^
Retroperitoneal bleeding	1/1061 (0.1)	0/458 (0.0)	1/603 (0.2)	^c^
Arteriovenous fistula	0/1061 (0.0)	0/458 (0.0)	0/603 (0.0)	^c^
Minor bleeding	77/1061 (7.3)	23/458 (5.0)	54/603 (9.0)	**.014**
Overall bleeding^d^	94/1061 (8.9)	25/458 (5.5)	69/603 (11.4)	**<.01**

Abbreviations: TFA, transfemoral access; TRA, transradial access.

Bold values highlight the statistical significance. ^a^ Values are n (%).

^b^ According to BARC classification.

^c^ No statistical test was performed because of low number of events.

^d^ Includes major and/or minor bleeding.

In the univariable analysis, TRA was associated with fewer MVC, major bleeding, minor bleeding, and overall bleeding (Table 4). After PS adjustment in the multivariable analysis, TRA turned out to be independently associated with lower rates of MVC, major bleeding, minor bleeding, and overall bleeding (Table 4). The PS model, with all variables included, is shown as supplemental material (supplement 1).

**Table 4.** Uni- and Multivariable Analysis for Transradial Access as Predictor for Major Vascular Complications and Bleeding.^a^

Outcome	Univariable	*P*	Multivariable^b^	*P*
Major vascular complications	0.21 (0.06-0.73)	**.014**	0.24 (0.07-0.83)	**.024**
Major bleeding	0.16 (0.04-0.70)	**.015**	0.21 (0.05-0.93)	**.040**
Minor bleeding	0.54 (0.33-0.89)	**.016**	0.49 (0.29-0.83)	**<.01**
Overall bleeding^c^	0.45 (0.28-0.72)	**<.01**	0.44 (0.27-0.72)	**<.01**

^a^ Values are odds ratios (ORs) with 95% CIs.

^b^ Adjusted for propensity score.

^c^ Includes major and/or minor bleeding.

### Efficacy Endpoints

**Figure 2.**
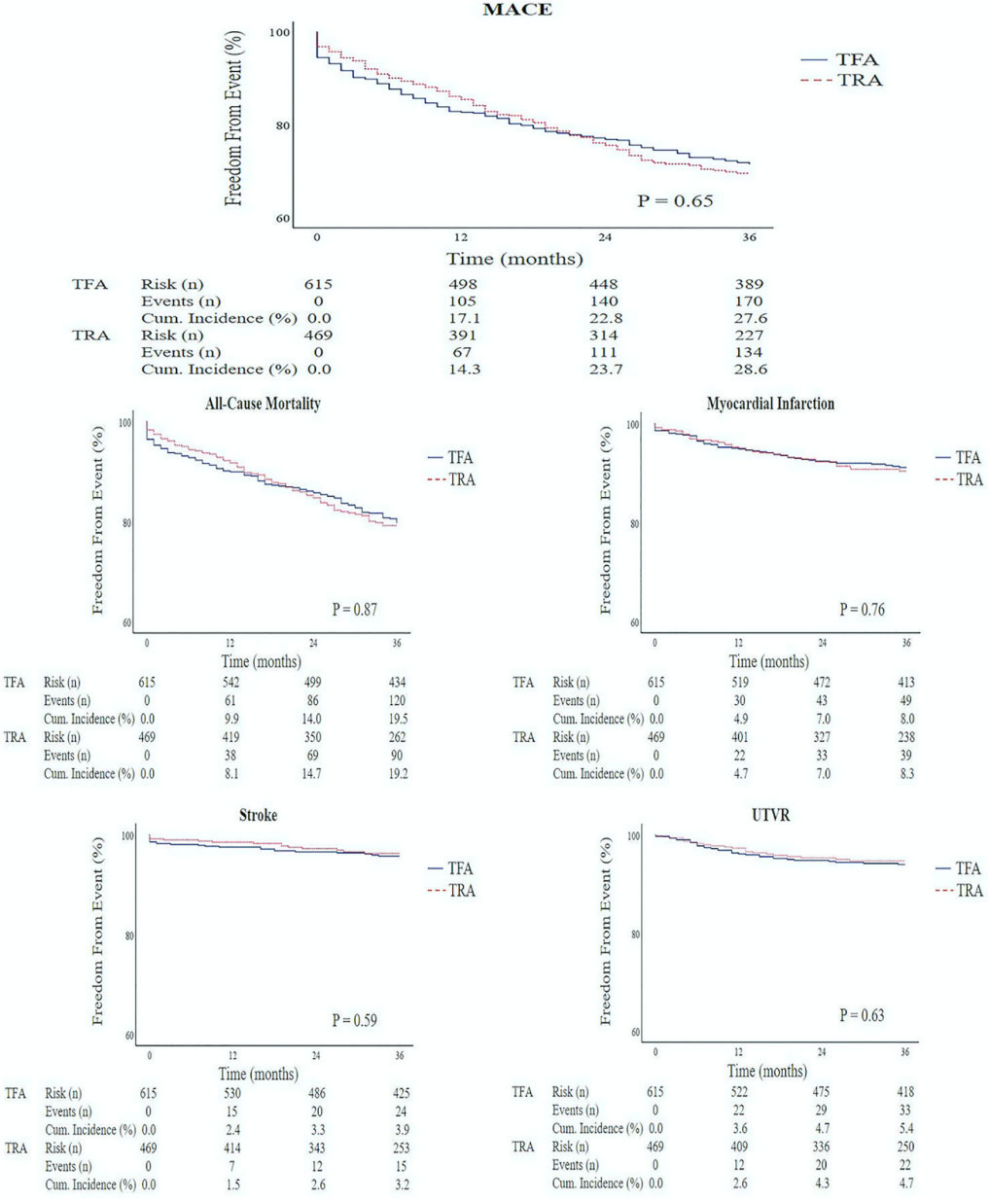
Long-term Kaplan-Meier curves for the composite primary efficacy endpoint (major adverse cardiovascular events) and each individual component. Survival plots (36 months) for transfemoral versus transradial access. MACE indicates major adverse cardiovascular events; TFA, transfemoral access; TRA, transradial access; UTVR, urgent target vessel revascularization.

At 36 months, the cumulative incidence of MACE was 28.6% (134 events) for the TRA group and 27.6% (170 events) for the TFA group, respectively. Kaplan-Meier curves for the TRA and TFA group showed no difference for the primary efficacy endpoint (*P* = .65; Figure 2). In addition, for the secondary efficacy endpoints (all-cause mortality, MI, stroke, and UTVR), no significant differences were observed.

## Discussion

This study compared TRA with TFA for CA and PCI in patients with prior CABG, in terms of short-term safety and long-term efficacy outcomes. The main findings are (1) TRA was associated with significantly fewer MVC at 30 days, (2) no difference was observed in MACE at 36 months between TRA and TFA, and (3) contrast use was significantly less with TRA.

Our study showed that TRA was associated with less MVC, which remained significant after PS adjustment. The significant difference in favor of TRA was mainly driven by a lower rate of major bleeding, despite a higher percentage of triple anticoagulation therapy. Our findings are opposite to the findings of the RCT by Michael et al,^15^ in which they compared TRA and TFA in 128 patients with prior CABG. The primary endpoint of the study was the volume of contrast use during the procedure but also vascular complications were recorded. They reported no difference in vascular complications and in-hospital major bleeding between both access sites, using the same BARC definitions. Possible explanations for this difference are the follow-up time, as follow-up stopped at hospital discharge in their study, and the small sample size, as it was not powered to find a difference in vascular complications or bleeding. A recently published RCT compared net procedure time (time from sheath introduction to procedure completion) for left-TRA with TFA in 150 patients with prior CABG and reported no difference for the secondary outcome of local vascular complications.^17^ However, as this was only a secondary endpoint, this study was probably not powered for this endpoint. Another recent, large retrospective trial that included 58 870 patients found similar results with our study.^10^ They showed that TRA was associated with fewer arterial complications and in-hospital major bleeding. However, they analyzed these in-hospital outcomes separately, while we used a composite outcome that included both vascular complications and major bleeding at 30 days. The only meta-analysis on this subject included 9 studies up to October 2015.^18^ TRA was associated with fewer access site complications, but definitions were different for each study. Summarizing these studies and with the current clinical practice in mind, TRA seems to be at least as safe as TFA in patients with prior CABG.

To our knowledge, this is the first study presenting long-term clinical outcomes for TRA and TFA in patients with prior CABG. The Kaplan-Meier curves did not differ significantly for long-term MACE and each single component of MACE. In the aforementioned study by Michael et al, in-hospital MACE was a secondary outcome, defined as all-cause mortality, MI, stroke, and UTVR.^15^ During the study period, none of these events occurred. This might be due to small sample size, short follow-up time, and the exclusion of old patients (age >90 years) and ST-elevation MI. Furthermore, there are some retrospective studies comparing clinical outcomes for both access sites. Two small studies did not find any difference for MACE at 1-year follow-up.^12,13^ The large retrospective study did not use MACE as a composite endpoint.^10^ However, the adjusted TRA and TFA Kaplan-Meier curves for 1-year mortality did not show any significant difference. In our study, 1-year clinical outcomes were not a study endpoint, but TRA and TFA Kaplan-Meier curves for this time period are provided as supplemental material (supplement 2). No significant differences for 1-year MACE, and each individual component of MACE, were observed among these access sites. Altogether, it seems that the choice for TRA or TFA in patients with prior CABG does not affect MACE.

In the present study, we show the transformation of TRA as preferred access site in this subset of patients, just as Kinnaird et al did in the United Kingdom.^10^ However, when compared with the overall population, in over 85.0% of PCI procedures performed in the Netherlands between 2017 and 2018, TRA was used.^19^ A possible explanation for the fact that TRA still lags behind in patients with prior CABG might be the ignorance of CABG anatomy in the acute setting through missing data (surgery reports). However, in our study CABG anatomy was known in the great majority of patients presenting with STEMI and access site did not differ significantly in these patients if CABG anatomy was unknown. In addition, imaging the left internal mammary artery is more effective through TFA and left TRA than through right TRA,^20^ but left TRA has its ergonomic disadvantages.^21^ Furthermore, potential disadvantages of TRA, such as higher crossover rates might explain the preference for TFA. In our study, arterial crossover occurred significantly more from TRA to TFA, than vice versa. The main reasons for crossover were not obtaining arterial access, inability to advance the catheter, and radial spasm. These issues were also described in the literature.^22–24^ Other studies also reported more arterial crossover with TRA,^12,13,15,17,18^ except for one study which found a slightly higher crossover rate from TFA to TRA.^11^ In our study, the higher arterial crossover rate from TRA to TFA did not lead to longer procedure duration in the TRA group, as procedure time was comparable for both access sites. Other more recent studies also observed no significant differences in procedure time between TRA and TFA.^11,12,17,18^

We found that radiation exposure (dose area product) was significantly lower with TRA compared with TFA. However, this might be more a reflection of refinement of radiation use over time during cardiac catheterization. For example, the use of fluoroscopy became the standard supportive tool for stent placement and balloon inflation instead of filming (cine). This is supported by the fact that there was no difference in fluoroscopy time. A recent prospective trial also showed no significant differences in radiation exposure and fluoroscopy time.^17^

Finally, contrast use in cardiac catheterizations remains an important topic and one should always minimize this, as its usage can lead to contrast-induced acute kidney injury. This serious complication has kidney-related consequences and may even affect long-term outcomes.^25^ The lower contrast volume use with TRA in the present study differs from the findings of Michael et al.^15^ They reported higher contrast use in the TRA group for CA and no difference in contrast use for PCI between both access sites. Furthermore, Rigattieri et al observed no difference between TRA and TFA.^18^ This discrepancy might be explained by the fact that our study covered a later time period in which TRA use and techniques had improved. The recent studies by Hirzallah et al and Dai et al support this assumption.^11,12^ Tsigkas et al found a nonsignificant difference in contrast use between both access sites.^17^

### Study Limitations

This study has some limitations. Firstly, it was a retrospective study. Selection bias and other forms of bias resulting from lack of randomization might have influenced results. To account for this, a PS model was used to correct for potential differences in characteristics of patients assigned to TRA or TFA.^26^ Secondly, underreporting of outcomes might have occurred due to suboptimal documentation by treating physicians and patients. Thirdly, the activated clotting time (ACT) was measured according to local protocol, but not systematically registered during most of the study period. Consequently, we were not able to present these data and could not adjust for a possible longer ACT in the TRA or TFA access group. Fourthly, the high rate of early CA and PCI after CABG is a noteworthy limitation, as establishing bleeding cause in these patients can be challenging. Finally, ultrasound guidance was not commonly used to obtain TFA during the study period. Nowadays, the use of this cotemporary technique is far more common and reduces vascular complications.^27^ Therefore, this is an important detail to take into account when interpreting these study results.

## Conclusion

In this retrospective study, the use of TRA for cardiac catheterizations in patients with prior CABG was associated with significantly fewer short-term MVC and contrast use, but not with a difference in long-term MACE, compared with TFA.

## Supplemental Material

Supplemental Material, sj-pdf-1-ang-10.1177_0003319720987351 - Cardiac Catheterizations in Patients With Prior Coronary Bypass Surgery: Impact of Access Strategy on Short-Term Safety and Long-Term Efficacy Outcomes Click here for additional data file. Supplemental Material, sj-pdf-1-ang-10.1177_0003319720987351 for Cardiac Catheterizations in Patients With Prior Coronary Bypass Surgery: Impact of Access Strategy on Short-Term Safety and Long-Term Efficacy Outcomes by Frederik T. Groenland, Jeroen M. Wilschut, Stijn C. van den Oord, Isabella Kardys, Roberto Diletti, Peter P. de Jaegere, Felix Zijlstra, Joost Daemen, Nicolas M. Van Mieghem and Wijnand K. den Dekker in Angiology

## References

[1] Roffi M Patrono C Collet JP, et al. 2015 ESC Guidelines for the management of acute coronary syndromes in patients presenting without persistent ST-segment elevation: Task Force for the Management of Acute Coronary Syndromes in Patients Presenting without Persistent ST-Segment Elevation of the European Society of Cardiology (ESC). Eur Heart J. 2016;37:267–315. 26320110 10.1093/eurheartj/ehv320

[2] Ibanez B James S Agewall S, et al. 2017 ESC Guidelines for the management of acute myocardial infarction in patients presenting with ST-segment elevation: The Task Force for the management of acute myocardial infarction in patients presenting with ST-segment elevation of the European Society of Cardiology (ESC). Eur Heart J. 2018;39:119–77. 28886621 10.1093/eurheartj/ehx393

[3] Knuuti J Wijns W Saraste A, et al. 2019 ESC Guidelines for the diagnosis and management of chronic coronary syndromes: The Task Force for the diagnosis and management of chronic coronary syndromes of the European Society of Cardiology (ESC). Eur Heart J. 2019;41:407–77. 10.1093/eurheartj/ehz425 31504439

[4] Jolly SS Yusuf S Cairns J, et al. Radial versus femoral access for coronary angiography and intervention in patients with acute coronary syndromes (RIVAL): a randomised, parallel group, multicentre trial. Lancet. 2011;377:1409–20. 21470671 10.1016/S0140-6736(11)60404-2

[5] Valgimigli M Gagnor A Calabro P, et al. Radial versus femoral access in patients with acute coronary syndromes undergoing invasive management: a randomised multicentre trial. Lancet. 2015;385:2465–76. 25791214 10.1016/S0140-6736(15)60292-6

[6] Kolkailah AA Alreshq RS Muhammed AM Zahran ME Anas El-Wegoud M Nabhan AF. Transradial versus transfemoral approach for diagnostic coronary angiography and percutaneous coronary intervention in people with coronary artery disease. Cochrane Database Syst Rev. 2018;4:CD012318. 29665617 10.1002/14651858.CD012318.pub2 PMC6494633

[7] Ferrante G Rao SV Jüni P, et al. Radial Versus Femoral Access for Coronary Interventions Across the Entire Spectrum of Patients With Coronary Artery Disease: A Meta-Analysis of Randomized Trials. J Am Coll Cardiol Interv. 2016;9:1419–34. 10.1016/j.jcin.2016.04.014 27372195

[8] Valgimigli M Frigoli E Leonardi S, et al. Radial versus femoral access and bivalirudin versus unfractionated heparin in invasively managed patients with acute coronary syndrome (MATRIX): final 1-year results of a multicentre, randomised controlled trial. Lancet. 2018;392:835–48. 30153988 10.1016/S0140-6736(18)31714-8

[9] Brilakis ES O’Donnell CI Penny W, et al. Percutaneous Coronary Intervention in Native Coronary Arteries Versus Bypass Grafts in Patients With Prior Coronary Artery Bypass Graft Surgery: Insights From the Veterans Affairs Clinical Assessment, Reporting, and Tracking Program. J Am Coll Cardiol Interv. 2016;9:884–93. 10.1016/j.jcin.2016.01.034 27085582

[10] Kinnaird T Anderson R Gallagher S, et al. Vascular Access Site and Outcomes in 58,870 Patients Undergoing Percutaneous Coronary Intervention With a Previous History of Coronary Bypass Surgery: Results From the British Cardiovascular Interventions Society National Database. J Am Coll Cardiol Interv. 2018;11:482–92. 10.1016/j.jcin.2017.12.020 29519382

[11] Hirzallah H Amro A Kusmic D, et al. Comparison of transradial and transfemoral approaches for coronary angiography and percutaneous intervention in patients with coronary bypass grafts. Cardiovasc Revasc Med. 2020;21:2–5. 30885499 10.1016/j.carrev.2019.03.002

[12] Dai Y Li C Zhang F, et al. Safety and Efficacy of Percutaneous Coronary Intervention via Transradial Versus Transfemoral Approach in Bypass Grafts. Angiology. 2018;69:136–42. 28602142 10.1177/0003319717711765

[13] He PY Yang YJ Qiao SB, et al. A comparison of the transradial and transfemoral approaches for the angiography and intervention in patients with a history of coronary artery bypass surgery: in-hospital and 1-year follow-up results. Chin Med J (Engl). 2015;128:762–7. 25758269 10.4103/0366-6999.152488 PMC4833979

[14] Han H Zhou Y Ma H, et al. Safety and feasibility of transradial approach for coronary bypass graft angiography and intervention. Angiology. 2012;63:103–8. 21571729 10.1177/0003319711408863

[15] Michael TT Alomar M Papayannis A, et al. A randomized comparison of the transradial and transfemoral approaches for coronary artery bypass graft angiography and intervention: the RADIAL-CABG Trial (RADIAL Versus Femoral Access for Coronary Artery Bypass Graft Angiography and Intervention). J Am Coll Cardiol Interv. 2013;6:1138–44. 10.1016/j.jcin.2013.08.004 24139930

[16] Mehran R Rao SV Bhatt DL, et al. Standardized bleeding definitions for cardiovascular clinical trials: a consensus report from the Bleeding Academic Research Consortium. Circulation. 2011;123:2736–47. 21670242 10.1161/CIRCULATIONAHA.110.009449

[17] Tsigkas G Makris A Tsiafoutis I, et al. The L-Record Study. J Am Coll Cardiol Interv. 2020;13:1014–6. 10.1016/j.jcin.2020.02.013 32327086

[18] Rigattieri S Sciahbasi A Brilakis ES, et al. Meta-Analysis of Radial Versus Femoral Artery Approach for Coronary Procedures in Patients With Previous Coronary Artery Bypass Grafting. Am J Cardiol. 2016;117:1248–55. 26892452 10.1016/j.amjcard.2016.01.016

[19] How does UK interventional practice compare with Europe? By Baumbach A. Published June 2019. Accessed January 7, 2021. Available at https://www.bcis.org.uk/wp-content/uploads/2019/06/Intervention-in-Europe2019_compressed.pdf

[20] Balaban Y Akbas MH Akbas ML Ozerdem A. Efficacy and Safety of “Coronary Artery Bypass Graft Angiography” with Right Transradial Access versus Left Transradial Access and Femoral Access: a Retrospective Comparative Study. Braz J Cardiovasc Surg. 2019; 34:48–56. 30810674 10.21470/1678-9741-2018-0270 PMC6385842

[21] Kado H Patel AM Suryadevara S, et al. Operator radiation exposure and physical discomfort during a right versus left radial approach for coronary interventions: a randomized evaluation. J Am Coll Cardiol Interv. 2014;7:810–6. 10.1016/j.jcin.2013.11.026 24954573

[22] Achenbach S Ropers D Kallert L, et al. Transradial versus transfemoral approach for coronary angiography and intervention in patients above 75 years of age. Catheter Cardiovasc Interv. 2008;72:629–35. 18798237 10.1002/ccd.21696

[23] Brueck M Bandorski D Kramer W Wieczorek M Holtgen R Tillmanns H. A randomized comparison of transradial versus transfemoral approach for coronary angiography and angioplasty. J Am Coll Cardiol Interv. 2009;2:1047–54. 10.1016/j.jcin.2009.07.016 19926042

[24] Roberts EB Rathore S Beaumont A, et al. Lesion complexity and angiographic outcomes in radial access percutaneous coronary intervention. J Interv Cardiol. 2008;21:555–61. 18973507 10.1111/j.1540-8183.2008.00399.x

[25] Uzunhasan I Yildiz A Arslan S, et al. Contrast-Induced Acute Kidney Injury Is Associated With Long-Term Adverse Events in Patients With Acute Coronary Syndrome. Angiology. 2017;68:621–6. 28660805 10.1177/0003319716676173

[26] Elze MC Gregson J Baber U, et al. Comparison of Propensity Score Methods and Covariate Adjustment: Evaluation in 4 Cardiovascular Studies. J Am Coll Cardiol. 2017;69:345–57. 28104076 10.1016/j.jacc.2016.10.060

[27] Sobolev M Slovut D Lee Chang A Shiloh A Eisen L. Ultrasound-guided catheterization of the femoral artery: A systematic review and meta-analysis of randomized controlled trials. J Invasive Cardiol. 2015;27:318–23. 26136279

