# Supplementary material for: Cardiac Catheterizations in Patients With Prior Coronary Bypass Surgery: Impact of Access Strategy on Short-Term Safety and Long-Term Efficacy Outcomes
Source: Angiology. 2021 Jan 19;72(5):465–73. doi: 10.1177/0003319720987351 (PMC8044620; doi:10.1177/0003319720987351)
Supplement: Supplemental Material, sj-pdf-1-ang-10.1177_0003319720987351 - Cardiac Catheterizations in Patients With Prior Coronary Bypass Surgery: Impact of Access Strategy on Short-Term Safety and Long-Term Efficacy Outcomes [file sj-pdf-1-ang-10.1177_0003319720987351.pdf]

Supplemental Material

Supplement 1 (additional table). Propensity score model for transradial access.

**Variable**	**Multivariable**	**p**
**Age**	1.01 (1.00-1.03)	0.07
**Male**	1.11 (0.80-1.54)	0.52
**Hypertension**	1.29 (0.91-1.81)	0.15
**Hypercholesterolemia**	1.16 (0.83-1.63)	0.40
**Diabetes**	0.99 (0.75-1.31)	0.96
**Family History**	0.85 (0.64-1.13)	0.25
**Current Smoker**	0.78 (0.49-1.25)	0.30
**Prior Stroke**	0.99 (0.68-1.42)	0.94
**Prior MI**	0.86 (0.65-1.12)	0.26
**Prior PCI**	0.86 (0.65-1.14)	0.30
**Peripheral Artery Disease**	0.89 (0.65-1.22)	0.47
**Chronic Kidney Disease**	0.84 (0.61-1.15)	0.27
**CABG Anatomy Known**	1.17 (0.54-2.56)	0.69
**LIMA Used**	1.05 (0.72-1.51)	0.81
**RIMA Used**	1.48 (0.95-2.30)	0.08
**SVG Used**	0.87 (0.56-1.34)	0.52
**CA/PCI <48 h Post-CABG**	0.30 (0.15-0.62)	**< 0.01**
**STEMI**	0.63 (0.38-1.04)	0.07
**PCI Performed**	1.63 (0.59-4.49)	0.35
**Sheath Size (6 French)**	0.41 (0.19-0.88)	**0.022**
**Native Vessel Treated**	0.66 (0.20-2.14)	0.48
**Graft Vessel Treated**	0.44 (0.13-1.48)	0.19

Supplement 1 (additional table). Propensity score model for transradial access (continuation).

Variable	Multivariable	p
**Multiple Vessels Treated**	0.52 (0.14-1.91)	0.33
**Lesion Class B2C**	0.80 (0.46-1.38)	0.43
**Implanted Stents**	0.79 (0.57-1.09)	0.16
**Total Stent Length**	1.01 (1.00-1.02)	0.20
**Procedure Time**	1.00 (1.00-1.01)	0.34
**Fluoroscopy Time**	1.01 (1.00-1.03)	0.09
**Radiation Exposure**	1.00 (1.00-1.00)	**< 0.01**
**Glycoprotein Inhibitor Used**	0.77 (0.39-1.52)	0.45
**Thienopyridine Derivate Used**	1.75 (1.07-2.86)	**0.025**

Values are odds ratios (ORs) with 95% confidence intervals (CIs).

CA = coronary angiography; CABG = coronary artery bypass graft; LIMA = left internal mammary artery; MI = myocardial infarction; PCI = percutaneous coronary intervention; RIMA = right internal mammary artery; STEMI = ST-elevation myocardial infarction; SVG = saphenous vein graft.

Supplement 2 (additional figure). 1-year Kaplan-Meier curves for the composite primary efficacy endpoint (major adverse cardiovascular events) and each individual component.

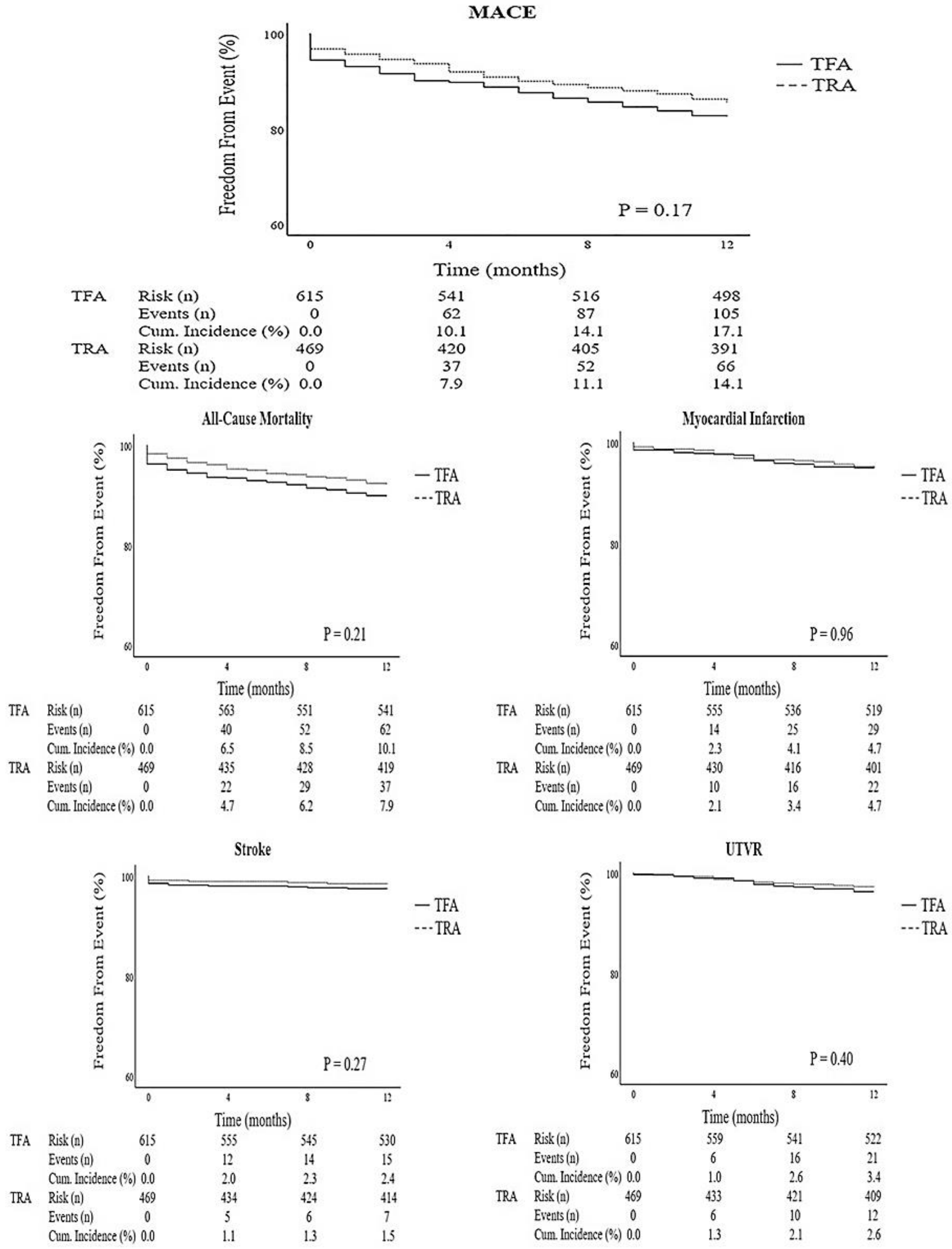

Survival plots (12 months) for transfemoral *vs* transradial access.

MACE = major adverse cardiovascular events; TFA = transfemoral access; TRA = transradial access; UTVR = urgent target vessel revascularization.
